# Supplementary figures and images for: Morphometric and microsatellite-based comparative genetic diversity analysis in Bubalus bubalis from North India
Source: PeerJ. 2021 Aug 11;9:e11846. doi: 10.7717/peerj.11846 (PMC8364325; doi:10.7717/peerj.11846)

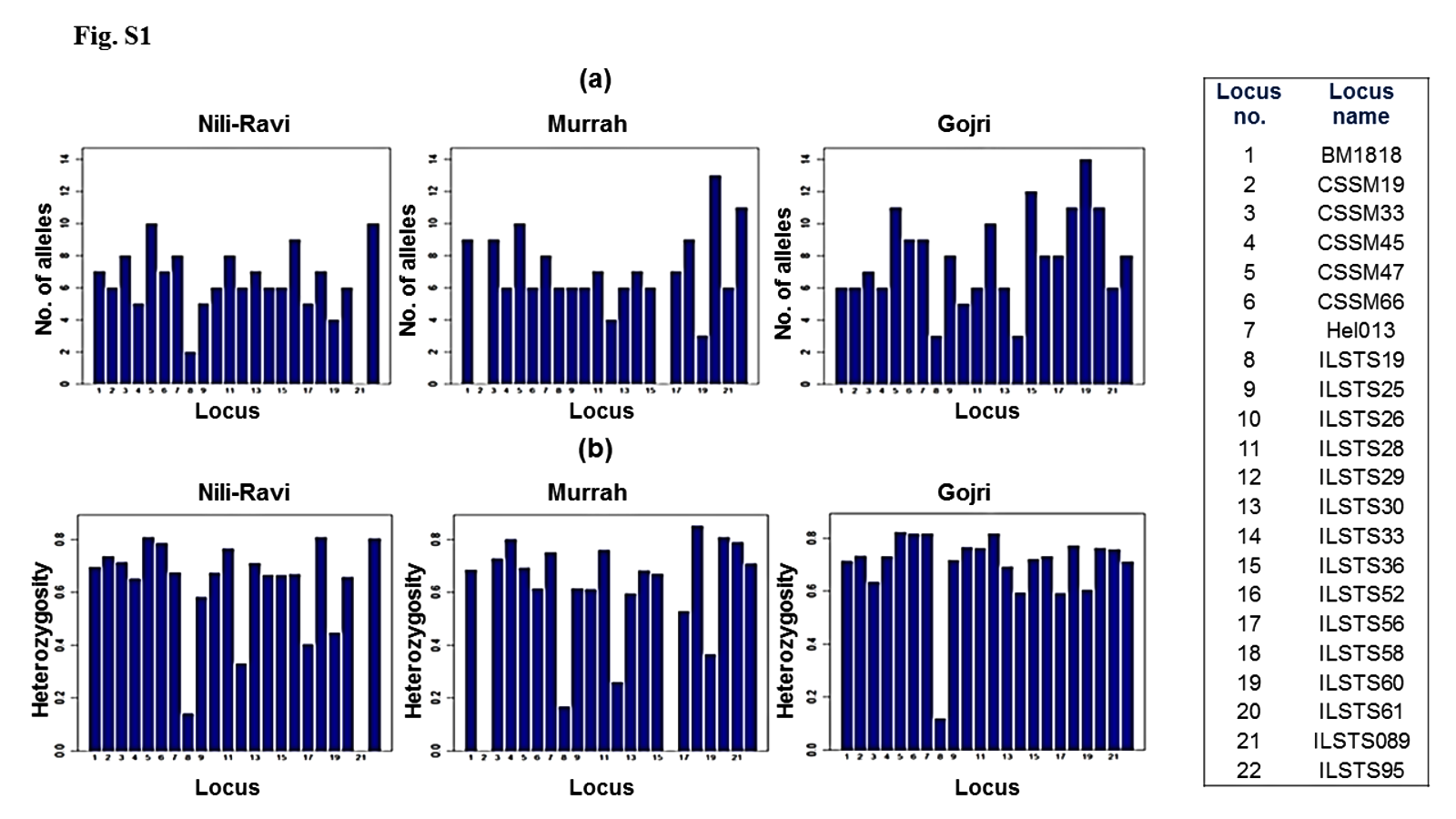

Supplement: Supplemental Information 2 — (a) Number of alleles (Na) per locus among the Nili-Ravi, Murrah and Gojri buffalo populations (b) Expected heterozygosity (He) per locus among the Nili-Ravi, Murrah and Gojri buffalo populations [file peerj-09-11846-s002.png]

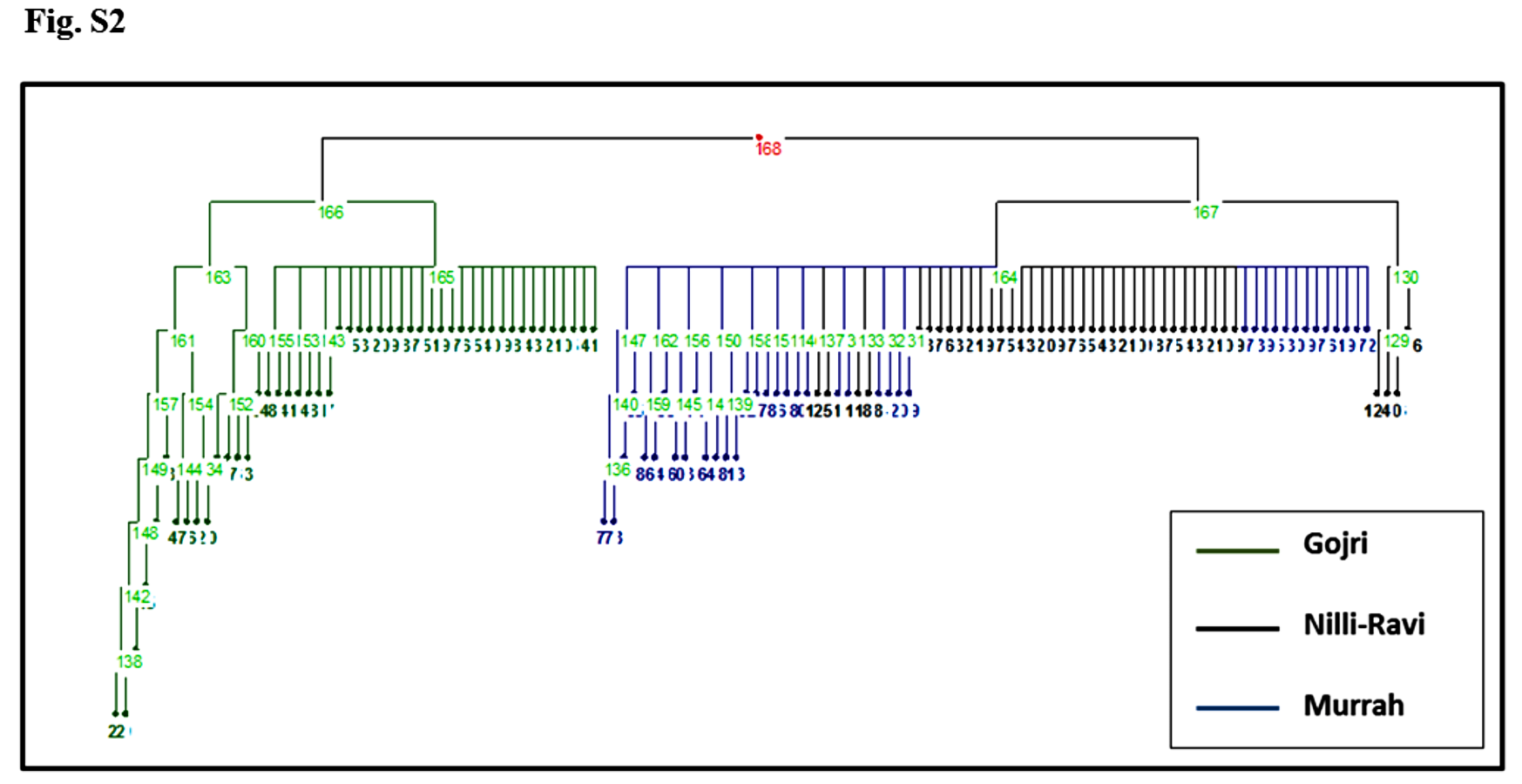

Supplement: Supplemental Information 3 [file peerj-09-11846-s003.png]

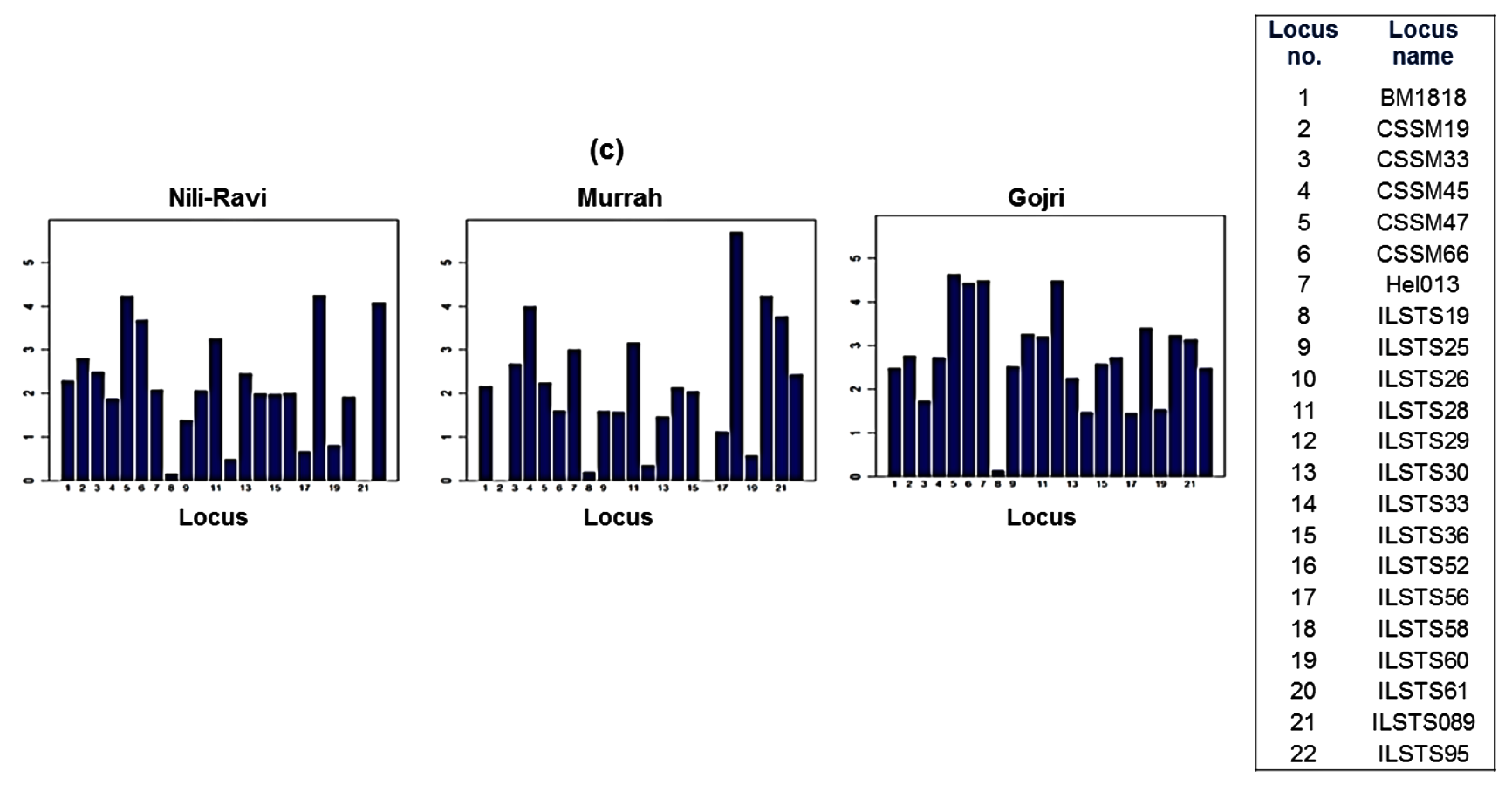

Supplement: Supplemental Information 4 — (c) Theta H (θH) obtained per locus among the Nili-Ravi, Murrah and Gojri buffalo populations [file peerj-09-11846-s004.png]
